# Muscular Atrophies in Joint Disease

**Published:** 1904-06-18

**Authors:** 


### Hospital Clinics.

### MUSCULAR ATROPHIES IN JOINT DISEASE.

The first and most natural suggestion which occurs to the mind when atrophy of the muscles in the neighbourhood of a diseased or injured joint is observed, is that such atrophy is the effect of disuse, and there can be little or no doubt that disuse is in many instances of this kind a factor of large influence. In, for example, a limb which has been fixed in a splint for some weeks the absence of muscular action and the consequent defective blood supply establish the physiological conditions under which malnutrition and wasting inevitably take place. But tissue nutrition, as is well known, is dependent not merely on tissue activity and adequate blood supply. It is in some subtle and penetrating fashion controlled by influences which take origin in the central nervous system and which are conveyed to the muscles and other organs by the peripheral nerves. Thus, in studying the subject of muscular atrophy in joint disease, it becomes necessary to consider whether it may not be the case that the wasting is due to some disturbance of this nervous nutritive, or so-called "trophic," control. At first sight such a suggestion seems highly improbable. The mechanisms in which the trophic force is originated and by which it is conveyed to the muscles are not, in a case of joint disease, apparently involved. Both the centres in the spinal cord and the nerves passing thence to the muscles are intact, and therefore, at least at first sight, it seems highly unlikely that prejudice to muscular nutrition can arise upon this side. It must be remembered, however, that the trophic centres in the spinal cord or elsewhere are, like other nervous ganglia, connected with afferent nervous pathways and that it is at least possible that nerve impressions travelling along these may reach and modify the activity of the trophic centres; and further that as a consequence of such modification the nutrition of more or less remote tissues may be disturbed. Again, it is possible to conceive, at least in theory, that afferent nerve impressions capable of influencing the activity of trophic nerve centres may take origin in disturbances existing in almost any part of the body, including presumably, therefore, the joints. That is to say, that defective nutrition or atrophy of the muscles or other tissues may be a reflex result, through the trophic centres, of inflammatory or other lesions affecting peripheral organs. Hence it is not sufficient in contemplating muscular atrophy in joint disease to say that as nerve centres and efferent nerve tracts are not involved in the diseased process the atrophy cannot be due to disturbance of the "trophic" nervous mechanism. It has to be remembered that the activities of these mechanisms may be modified by peripheral irritation conveyed along afferent nerve paths, and it is at least conceivable that joint disease may be competent to institute such peripheral irritation. Therefore, at least in theory, muscular atrophy in joint disease may be due, not to disuse, but to a reflex nervous influence, the starting point of which is to be found in the joint itself. What evidence may be found to suggest that such a state of matters actually exists in practice? First is the fact that a joint lesion may be followed with great promptness by evidences of atrophy. The wasting is not necessarily postponed for weeks or months; it may be appreciated within a few days, or even hours after the development of the joint condition. Obviously, here there is not sufficient time for the element of disease to come into operation. Some other cause, and one capable of swift operation, must therefore be invoked. Again, as was pointed out by Charcot, the muscular atrophies which attend joint disease do not affect all the muscles equally; they display, rather, a marked tendency to involve the extensor groups. Disuse, in such circumstances, obviously cannot be the cause, or certainly cannot be the sole cause, of the atrophy. Further, the degree of wasting is not proportional to the extent of the joint lesion, or to the measure of inactivity produced by such lesion. In some instances a very serious joint condition, with considerable and prolonged restraint of movement, may produce but moderate wasting, whilst, on the other hand, there are cases in which even a slight and easily cured sprain will be followed by extreme atrophy. These considerations taken together effectively dispose of disuse as an adequate explanation of the muscular wasting which often attends lesions of the joints, and when considered in relation to the known effects which may be produced in muscles by lesions of the central nervous system strongly support the view that such wasting finds its interpretation in a defect or other abnormal quality of the trophic nerve influence. And that such defect can originate in lesions of the joints is not only suggested by such clinical experiences as those above referred to, but is further demonstrated by experiments which have shown that a synovitis artificially induced in a dog by the injection of solution of ammonia may be promptly followed by extreme muscular wasting in the neighbourhood of the affected joint. There is thus an abundant basis for the conclusion that a joint lesion may, through the irritation it produces in peripheral nerve endings, modify the activity of trophic centres in the central nervous system, and that one of the results of this modification may appear in the shape of muscular atrophy.

Sir James Paget, many years ago, when treating of the arthritic complaints met with in hysterical patients, dealt with this question of muscular atrophy and discussed it as one factor having value in the diagnosis of these complaints. His proposition was that "if a joint has long been very painful, and yet there is no wasting of the muscles near it, it is not inflamed." The question is therefore not divorced from practical considerations. Another point on which it touches is the immediate cause of the condition of the joints in the disease known by such names as rheumatoid arthritis, arthritis deformans, osteo-arthritis, etc. Muscular atrophies are, of course, frequent and prominent facts in this disease, and their occurrence has been used to support the theory that the cause of the joint changes is to be found in disturbed function of the spinal centres. Thus it is argued that such disturbance can undoubtedly produce joint lesions, as seen for example in the development of a Charcot's joint in the course of tabes dorsalis; that muscular atrophies may arise from the same cause; and that therefore the existence of muscular atrophies with joint affections in rheumatoid arthritis supports the view that the articular conditions are equally with the muscular atrophies of spinal origin. But from what has already been said it will be observed that this reasoning is inconclusive. It may be admitted that the muscular atrophies are of spinal origin without in the least admitting that the joint changes have a similar explanation. For, from what has already been stated, it is manifest that changes in the joints, from whatever cause arising, may produce through the trophic nervous mechanism muscular atrophy, and that therefore the occurrence of muscular atrophies in rheumatoid arthritis is no proof that the joint lesions in this disease arise from structural or functional changes in the spinal cord. They may take origin from an entirely different source, and yet to an equal extent be accompanied by muscular atrophies. In short, whatever may be advanced from other sources in support of the theory that the joint lesions of rheumatoid arthritis are trophic or spinal in origin, the existence of muscular atrophies in the disease lends no aid to that theory. Such atrophies, as is the case with many joint lesions other than those of rheumatoid arthritis, are secondary consequences of, not concurrent events with, the joint lesions, and are produced through a reflex mechanism, the initial change in which is aroused by the joint disease itself.

